# Impaired glucose metabolism reduces the neuroprotective action of adipocytokines in cognitively normal older adults with insulin resistance

**DOI:** 10.18632/aging.203668

**Published:** 2021-11-03

**Authors:** Karel M. Lopez-Vilaret, Jose L. Cantero, Marina Fernandez-Alvarez, Miguel Calero, Olga Calero, Mónica Lindín, Montserrat Zurrón, Fernando Díaz, Mercedes Atienza

**Affiliations:** 1 Laboratory of Functional Neuroscience, Universidad Pablo de Olavide, Seville, Spain; 2 CIBERNED, Network Center for Biomedical Research in Neurodegenerative Diseases, Madrid, Spain; 3 Chronic Disease Programme, Instituto de Salud Carlos III, Madrid, Spain; 4 Cognitive Neuroscience Laboratory, Universidade de Santiago de Compostela, Santiago de Compostela, Spain

**Keywords:** adiponectin, leptin, cognitive function, cortical thickness, metabolism

## Abstract

Evidence suggests that aging-related dysfunctions of adipose tissue and metabolic disturbances increase the risk of diabetes and metabolic syndrome (MtbS), eventually leading to cognitive impairment and dementia. However, the neuroprotective role of adipocytokines in this process has not been specifically investigated. The present study aims to identify metabolic alterations that may prevent adipocytokines from exerting their neuroprotective action in normal ageing. We hypothesize that neuroprotection may occur under insulin resistance (IR) conditions as long as there are no other metabolic alterations that indirectly impair the action of adipocytokines, such as hyperglycemia. This hypothesis was tested in 239 cognitively normal older adults (149 females) aged 52 to 87 years (67.4 ± 5.9 yr). We assessed whether the homeostasis model assessment-estimated insulin resistance (HOMA-IR) and the presence of different components of MtbS moderated the association of plasma adipocytokines (i.e., adiponectin, leptin and the adiponectin to leptin [Ad/L] ratio) with cognitive functioning and cortical thickness. The results showed that HOMA-IR, circulating triglyceride and glucose levels moderated the neuroprotective effect of adipocytokines. In particular, elevated triglyceride levels reduced the beneficial effect of Ad/L ratio on cognitive functioning in insulin-sensitive individuals; whereas under high IR conditions, it was elevated glucose levels that weakened the association of the Ad/L ratio with cognitive functioning and with cortical thickness of prefrontal regions. Taken together, these findings suggest that the neuroprotective action of adipocytokines is conditioned not only by whether cognitively normal older adults are insulin-sensitive or not, but also by the circulating levels of triglycerides and glucose, respectively.

## INTRODUCTION

Obesity is a growing worldwide health problem that promotes cellular senescence and accelerates ageing [1]. Besides affecting physical status, obesity impacts cognitive functioning and mental health across the lifespan, leading to functional decline and reduced quality of life [2], an association that becomes mainly evident in people aged over 64 years [3].

Obesity and ageing, in addition to sharing an abnormal fat accumulation in visceral adipose depots and ectopic tissues (i.e., lipotoxicity) and progressive white adipose tissue dysfunction, induce a chronic low-grade inflammatory response that is partially responsible for decreased insulin sensitivity [4, 5]. The increased adiposity associated with obesity and the redistribution of adipose tissue that occurs during ageing are accompanied by decreased adiponectin concentrations and increased leptin levels, which may reflect leptin resistance [6]. These changes in circulating adipocytokine levels also contribute to decreased insulin sensitivity by reducing their ability to activate different insulin signaling pathways [7].

Insulin, adiponectin and leptin have receptors distributed throughout the brain to support their neuroprotective function [8]. Insulin fulfills this role by regulating glutamate and GABA levels, promoting glycogen storage in astrocytes, and inhibiting neuronal necrosis and apoptosis [9, 10]. Although the mechanisms responsible for the neuroprotective action of adiponectin and leptin remain to be elucidated, both have been implicated in improving cognitive function in normal ageing, and in reducing the incidence of cerebrovascular diseases, cognitive decline and dementia [11–20]. However, a few studies have either reported results in the opposite direction [21–24] or have found no relationship [25–27].

One possible explanation for these inconsistencies may be the study of adiponectin and leptin in isolation instead of using the balance between both of them (i.e., the Ad/L ratio), which is considered a surrogate biomarker of adipose tissue functionality [28, 29]. This hypothesis is supported by a stronger association between the Ad/L ratio and IR compared to that shown by adiponectin or leptin alone [30], and by studies showing that the lower the Ad/L ratio the higher the risk of metabolic disturbances [31] and the greater the chronic low-grade inflammation [32]. To our knowledge, only a couple of studies to date have reported an association between the Ad/L ratio and cognitive functioning in dementia-free older adults, but results again went in opposite directions [13, 24].

The lack of consensus regarding the relationship between circulating adipocytokines and cognitive functioning in ageing may also result from the complex communication that the adipose tissue maintains with the brain and other organs to facilitate the control of insulin and glucose [33]. Although fully functional adipose tissue is required for the maintenance of insulin sensitivity, the presence of systemic IR may reflect different degrees of dysfunction of the adipose tissue and other tissues involved in insulin signaling pathways, depending on the level of chronic low-grade inflammation and/or the concurrent presence of hyperglycemia, hypertriglyceridemia, and other metabolic disturbances typically associated with obesity, type-2 diabetes, and MetS. Therefore, to understand the relationship between adipocytokines, brain integrity and cognitive functioning, it may be necessary to consider the potential metabolic deregulations that condition this association. We hypothesize that adipocytokines may continue to exert their neuroprotective effect under conditions of elevated HOMA-IR. However, neuroprotection may be severely diminished when glucose metabolism is deregulated, as this would indicate that adipose tissue function [34] and insulin responsiveness [35, 36] are increasingly impaired.

Chronic low-grade inflammation, IR, and hyperglycemia induce oxidative stress [37], which in turn has been linked to grey and white matter atrophy [38–40] and cognitive impairment [41]. Given that the prefrontal cortex becomes particularly vulnerable to the ageing process [42] and to oxidative stress during ageing [43], we also expected that the moderating influence of IR and metabolic alterations on the neuroprotective effect of adipocytokines would be manifested mainly in the prefrontal lobe, leading to changes in cortical thickness.

## RESULTS

### Moderating role of HOMA-IR and MetS components on the adipocytokine-cognition relationship

The hypothesis about the moderating effect of HOMA-IR and different MetS components on the relationship between adipocytokines and cognitive functioning in ageing was tested in a sample of 239 cognitively unimpaired older adults. Demographic, anthropometric and metabolic measures are shown in Table 1, for the total sample and stratified by sex.

**Table 1:** Demographic, anthropometric and metabolic measures.

	**Total sample (N = 239)**	**Females (n = 149)**	**Males (n = 90)**
Age (years)	67.4 ± 5.9	66.7 ± 6.2	68.5 ± 5.1
Education (years)	10.2 ± 5.2	10.3 ± 5.4	10.0 ± 4.9
BMI (kg/m^2^)	27.5 ± 3.8	27.3 ± 3.8	27.9 ± 3.7
Waist circumference (cm)	91.5 ± 11.4	89.3 ± 11.2	95.0 ± 10.8
Heart rate (ppm)	66.2 ± 9.9	67.1 ± 9.2	64.7 ± 11.0
CRF (ml min^-1^ Kg^-1^)	8.1 ± 2.2	7.1 ± 2.0	9.5 ± 1.8
Systolic blood pressure (mmHg)	133.3 ± 20.2	132.1 ± 19.6	135.4 ± 21.1
HDL (mmol/l)	1.6 ± 0.4	1.6 ± 0.4	1.5 ± 0.5
Triglycerides (mmol/l)	1.3 ± 0.7	1.3 ± 0.7	1.3 ± 0.6
Glucose (mmol/l)	5.7 ± 0.8	5.6 ± 0.7	5.7 ± 1.0
Insulin (pmol/l)	56.0 ± 31.0	53.3 ± 28.4	60.3 ± 34.6
HOMA-IR	2.4 ± 1.5	2.3 ± 1.4	2.6 ± 1.6
Adiponectin (ng/ml)	6.6 ± 2.2	6.0 ± 2.1	7.6 ± 2.0
Leptin (ng/ml)	20.6 ± 16.3	25.2 ± 15.9	12.9 ± 13.9
Ad/L ratio	0.62 ± 0.66	0.37 ± 0.34	1.03 ± 0.83
*Metabolic syndrome components*			
Abdominal obesity (no/yes)	123 / 116	86 / 63	37 / 53
Hypertension (no/yes)	86 / 153	55 / 94	31 / 59
Hyperglycemia (no/yes)	111 / 128	67 / 82	44 / 46
Hypertriglyceridemia (no/yes)	159 / 80	98 / 51	61/ 29
Low HDL (no/yes)	175 / 64	111 / 38	64 / 26
Metabolic syndrome (no/yes)	134 / 105	85 / 64	49 / 41

All continuous variables are expressed as mean ± SD. CRF, cardiorespiratory fitness.

To test the above-mentioned hypothesis, we applied the frequentist approach in R [44] as well as the Bayesian approach in JASP [45]. We built 30 models as a function of the different adipocytokines (i.e., adiponectin, leptin and Ad/L ratio), of the different MetS components (i.e., abdominal obesity, hypertension, hyperglycemia, hypertriglyceridemia, and low HDL), and according to whether only nuisance predictors were included (i.e., main effects and two-way interactions derived from the interaction between adipocytokines, HOMA-IR, and MetS component) or whether they also contained the predictor of interest (i.e., the interaction between adipocytokines, HOMA-IR, and MetS component). After checking that assumptions were not violated for any statistical approach, that there were no influential cases as indicated by the residuals vs. Leverage plot, and that there were no multicollinearity problems (i.e., variable inflation factor < 5), we proceeded to determine if the contribution of the three-way interaction was over and above the contribution from the nuisance predictors.

The MetS component of interest in each model was included as categorical factor in the three-way interaction term, while all other MetS components (continuous variables) were treated as covariates together with cardiorespiratory fitness (CRF), body mass index (BMI), age, sex, years of education, adiponectin or leptin when one of them was included in the three-way interaction term, as well as the two-way interaction terms (i.e., adipocytokine × HOMA-IR; adipocytokine × MetS component; HOMA-IR × MetS component).

**Figure 1:**
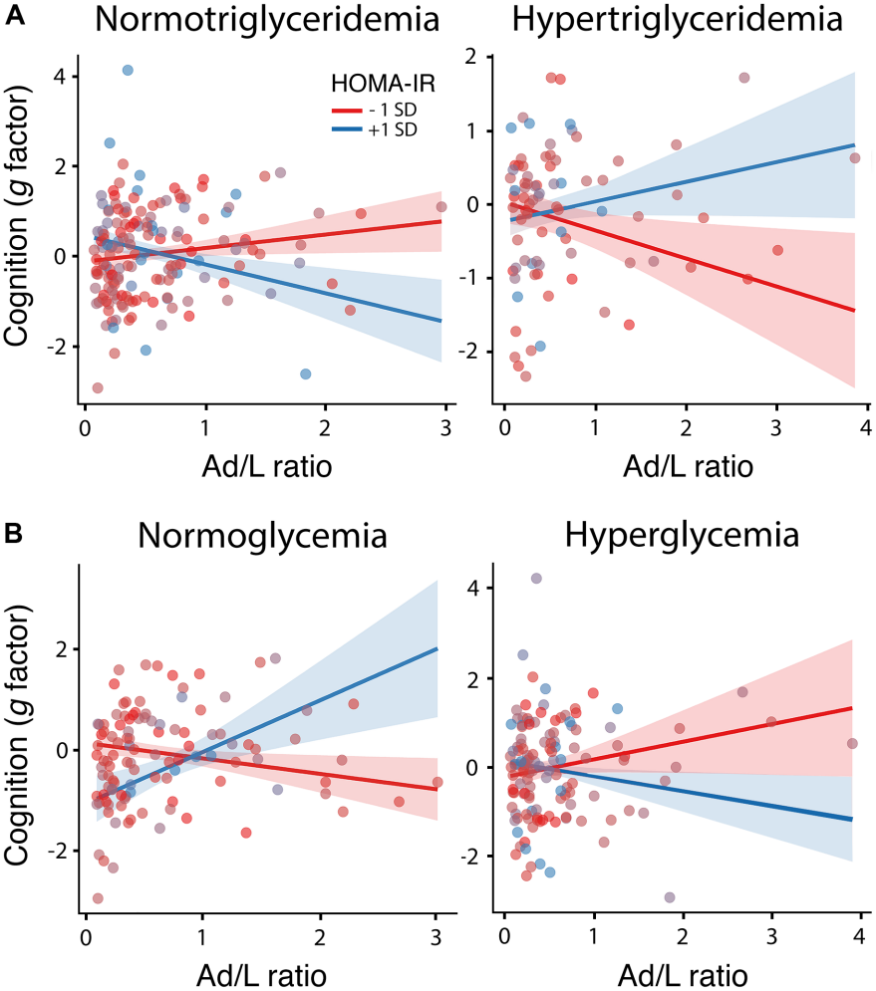
**Moderating role of HOMA-IR, triglyceridemia and glycemia in the association between the Ad/L ratio and cognition.** Association of the Ad/L ratio with cognition at 1 SD below and above the mean of HOMA-IR for participants showing normal levels of triglycerides (left panel) and hypertriglyceridemia (right panel) (**A**) or normal blood glucose levels (left panel) and hyperglycemia (right panel) (**B**) after adjustment of the remaining MtbS components, CRF, BMI, age, sex and years of education. The shaded areas reflect the confidence intervals (95%) for the fitted values.

The ANOVAs applied to compare the null and experimental models revealed a significant three-way interaction of the Ad/L ratio and HOMA-IR with the triglyceride (F_1,222_ = 9.1, p = 0.003, δ = 2.44, CI_0.95_ [0.32 4.56]) and glucose component of MetS (F_1,222_ = 8.9, p = 0.003, δ = -2.95, CI_0.95_ [-4.83 -1.09]). In the first case, the alternative hypothesis was approximately 18 times more likely than the null hypothesis (BF_10_ = 17.65), and in the second case 11 times more likely (BF_10_ = 11.11). Tables 2, 3 show the estimates, t-values and p-values for each parameter of these models. The Ad/L ratio was positively associated with cognitive functioning for decreased levels of HOMA-IR (i.e., at 1 SD below the mean) if triglyceride levels were normal, and for elevated levels of HOMA-IR (i.e., at 1 SD above the mean) if glucose levels were normal. These results are illustrated in Figure 1A, 1B, respectively.

**Table 2:** Three-way interaction between HOMA-IR, Ad/L ratio and triglycerides.

**Predictors**	**Model 1**			**Model 2**		
	***Estimate***	***t***	***p***	***Estimate***	***t***	***p***
Intercept	2.62	2.20	0.029	2.46	2.10	0.037
HOMA-IR	0.02	0.20	0.844	-0.01	-0.09	0.928
Ad/L ratio	0.01	0.10	0.919	-0.07	-0.73	0.468
Triglycerides	-0.14	-1.08	0.280	-0.13	-1.02	0.310
Glucose	-0.002	-0.46	0.646	-0.002	-0.46	0.650
Systolic blood pressure	0.003	0.94	0.347	0.004	1.15	0.250
HDL	-0.01	-1.84	0.067	-0.01	-1.68	0.095
Waist circumference	0.01	0.79	0.431	0.01	1.12	0.266
Cardiorespiratory fitness	0.003	0.07	0.941	0.01	0.24	0.808
Body mass index	-0.02	-0.86	0.389	-0.04	-1.30	0.196
Age	-0.05	-4.52	<.001	-0.05	-4.56	<.001
Sex	0.32	2.05	0.042	0.32	2.05	0.042
Education (years)	0.07	5.81	<.001	0.07	5.88	<.001
HOMA-IR*Ad/L	-0.04	-0.43	0.671	-0.32	-2.47	0.014
HOMA-IR*Triglycerides	0.01	0.09	0.925	0.08	0.66	0.512
Ad/L*Triglycerides	-0.02	-0.15	0.878	0.03	0.27	0.785
HOMA-IR*Ad/L*Triglycerides				0.53	3.02	0.003
Observations		239			239	
Min-max residuals		-2.81 4.59			-2.54 4.49	
R^2^		0.28			0.31	
Adjusted R^2^		0.24			0.26	
Residual Std. Error		0.87			0.86	
F Statistic		5.90			6.30	

**Table 3:** Three-way interaction between HOMA-IR, Ad/L ratio and fasting glucose.

**Predictors**	**Model 1**			**Model 2**		
	***Estimate***	***t***	***p***	***Estimate***	***t***	***p***
Intercept	2.43	2.20	0.029	2.29	2.11	0.036
HOMA-IR	-0.10	-0.81	0.421	-0.19	-1.55	0.122
Ad/L ratio	-0.003	-0.02	0.981	0.24	1.71	0.090
Glucose	0.24	1.89	0.061	0.30	2.39	0.018
Triglycerides	-0.001	-0.74	0.459	-0.001	-0.86	0.391
Systolic blood pressure	0.003	1.07	0.284	0.003	0.99	0.323
HDL	-0.01	-2.17	0.031	-0.01	-1.93	0.055
Waist circumference	0.01	0.77	0.441	0.01	0.85	0.398
Cardiorespiratory fitness	0.005	0.16	0.877	0.01	0.19	0.850
Body mass index	-0.03	-0.99	0.325	-0.02	-0.92	0.359
Age	-0.05	-4.60	<.001	-0.05	-4.75	<.001
Sex	0.35	2.26	0.025	0.35	2.26	0.025
Education (years)	0.07	5.87	<.001	0.07	5.98	<.001
HOMA-IR*Ad/L	-0.04	-0.34	0.735	0.44	2.32	0.021
HOMA-IR*Glucose	0.09	0.63	0.527	0.14	0.97	0.334
Ad/L*Glucose	-0.004	-0.03	0.978	-0.21	-1.40	0.163
HOMA-IR*Ad/L*Glucose				-0.67	-2.99	0.003
Observations		239			239	
Min-max residuals		-2.93 4.54			-2.57 4.50	
R^2^		0.29			0.32	
Adjusted R^2^		0.24			0.27
Residual Std. Error		0.87			0.85	
F Statistic		6.15			6.53	

The moderating effect of Ad/L ratio, HOMA-IR and triglycerides could be attributed to leptin, because when this adipocytokine was included in the interaction term, the three-way interaction was significant (F_2,221_ = 4.91, p = 0.027), although the strength of evidence was smaller than that of the Ad/L ratio (BF_10_ = 3.13) and the size effect was not significant (δ = -0.90, CI_0.95_ [-2.25 0.44]). None of the remaining three-way interactions were significant. In all cases, the Bayesian inference revealed absence of evidence (0.34 < BF_10_ < 1.48).

### Moderating role of HOMA-IR and fasting blood glucose/triglycerides on the relationship between Ad/L ratio and cortical thickness

Having determined that the association between the Ad/L ratio and cognitive functioning is moderated by HOMA-IR and by glucose and triglyceride levels, we wanted to assess whether these predictors also moderated the association of Ad/L with cortical thickness.

**Figure 2:**
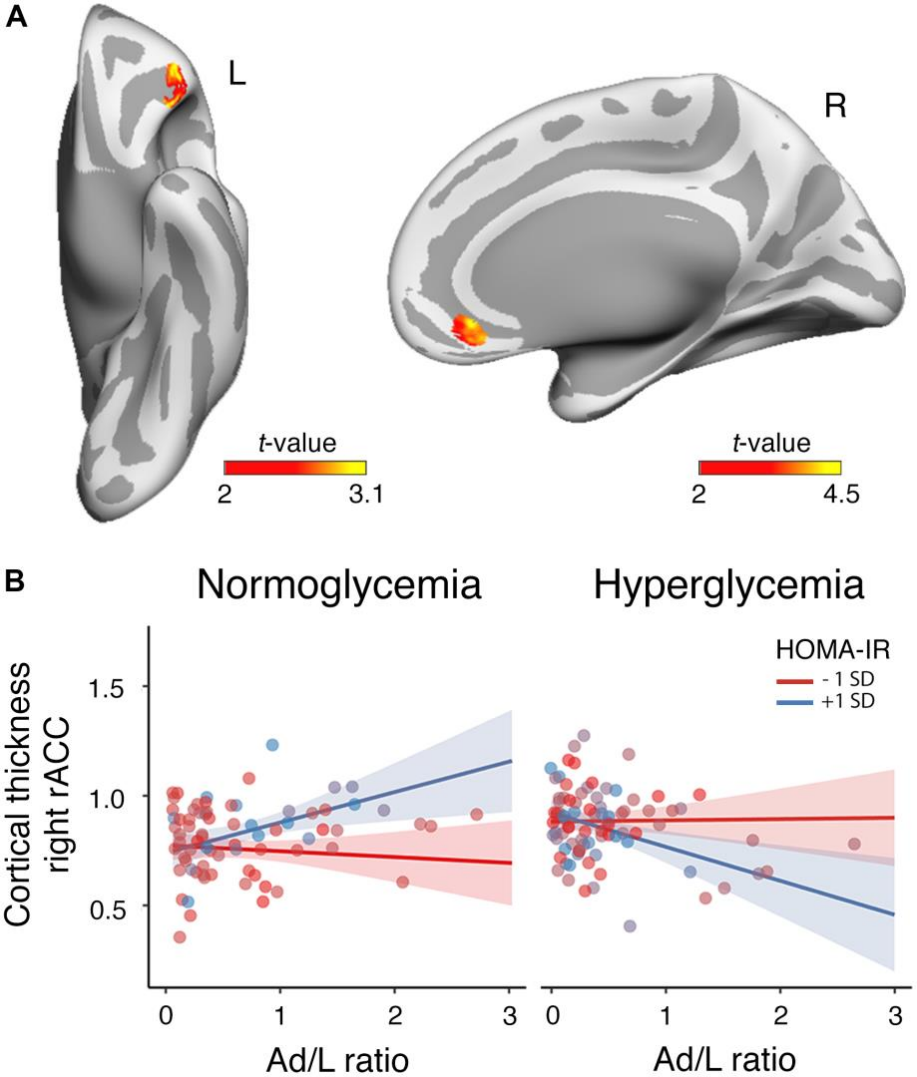
**Moderating role of HOMA-IR and glycemia in the association between the Ad/L ratio and cortical thickness.** (**A**) Results of the three-way interaction (Ad/L ratio × HOMA-IR × glycemia) after adjustment for the remaining MtbS components, CRF, BMI, age, sex and years of education. Significant t statistic values were projected into the inflated cortical surfaces (L: left; R: right). (**B**) Scatterplot to represent the post-hoc of the three-way interaction effect shown in panel (**A**). It shows the association of the Ad/L ratio with cortical thickness in the rostral anterior cingulate (rACC) of the right hemisphere at 1 SD below and above the mean of HOMA-IR for participants showing normal blood glucose levels (left panel) and hyperglycemia (right panel). The shaded areas reflect the confidence intervals (95%) for the fitted values.

The ANCOVA based on frequentist inference revealed that the interaction between HOMA-IR and serum glucose levels moderated the association of the Ad/L ratio with cortical thickness in the left lateral orbitofrontal cortex (R^2^ = 0.04, F_max_ = 97.76, p_cluster-corrected_ = 0.03, δ = 2.15 CI_0.95_ [0.49 3.80]) and the right rostral anterior cingulate cortex (R^2^ = 0.06, F_max_ = 7.02, p_cluster-corrected_ = 0.04, δ = 3.66 CI_0.95_ [2.03 5.30]). Indeed, the evidence was very strong in the left lateral orbitofrontal cortex (BF_10_ = 69.37) and extreme in the right anterior cingulate (BF_10_ = 178.12). These results are illustrated in Figure 2A. The positive association of the Ad/L ratio with cortical thickness in the right rostral anterior cingulate cortex was evident for elevated HOMA-IR values as long as participants were normoglycemic (Figure 2B). The assessment of the three-way interaction between Ad/L, HOMA-IR and triglycerides on cortical thickness did not yield significant results.

### Relationship between adipocytokines-related changes in cortical thickness and cognitive functioning

The Ad/L ratio moderated the relationship between thickness in the right rostral anterior cingulate cortex and cognitive functioning. The ANOVA applied to compare the interactive and additive model showed a significant two-way interaction (R^2^ = 0.32, F_2,224_ = 8.16, p = 0.005). In particular, cortical thickness in the right anterior cingulate cortex was more positively associated with cognition at 1 SD above the mean of the Ad/L ratio than at 1 SD below the mean (ρ = 0.72 CI_0.95_ [0.18 1.24]). The Bayesian analysis provided moderate evidence for the alternative hypothesis (BF_10_ = 6.43).

## DISCUSSION

The main objective of this study was to determine whether IR assessed by HOMA-IR together with other metabolic alterations associated with obesity, type-2 diabetes and MetS moderated the hypothesized neuroprotective action of adipocytokines in cognitively normal older adults. Our results suggest that the neuroprotective effect of adipocytokines is conditioned not only by the severity of IR but also by glucose and triglyceride metabolism. In particular, the results indicated that the Ad/L ratio is associated with better cognitive functioning and increased thickness of regions in the prefrontal cortex under conditions of elevated HOMA-IR, provided that glucose metabolism is under control. Although insulin-sensitive participants also benefited from adipocytokines at the cognitive level, this effect disappeared in those with hypertriglyceridemia.

### Hypertriglyceridemia reduces the neuroprotective action of the Ad/L ratio under high insulin sensitivity conditions

Among the main causes of elevated triglycerides in individuals with insulin sensitivity are obesity and adipose tissue dysfunction through mechanisms including chronic low-grade inflammation, increased lipolysis and altered adipocytokine production [33]. Our results suggest that hypertriglyceridemia reduces the cognitive benefit of adipocytokines in older adults without cognitive impairment and with high insulin sensitivity. Given that hypertriglyceridemia is recognized as an independent risk factor for atherosclerotic cardiovascular disease [46], one could think of vascular damage as a possible cause of the reduced beneficial effect of adipocytokines on cognitive functioning in ageing. Contrary to this hypothesis, fasting triglyceride levels were found to be inversely associated with executive function in non-demented community dwelling older adults, even after controlling for vascular risk factors such as LDL cholesterol, total cholesterol, ApoE4 status and cerebrovascular injury revealed by white matter microstructure damage [47].

Alternatively, the undesirable effect of triglycerides on the positive association between the Ad/L ratio and cognition could be due to their ability to penetrate the blood-brain barrier (BBB) and induce central leptin and insulin receptor resistance [48], two phenomena increasingly associated with ageing-related cognitive decline and Alzheimer’s disease (AD) [10, 49, 50]. There is also the possibility that elevated triglyceride levels are suggestive of increased chronic low-grade inflammation [51], which in turn can cause damage of the BBB [52] and neuroinflammation that ultimately leads to impaired cognitive function [53]. As neuroinflammation can occur with varying degrees of severity [54], it is possible that the associated functional damage may not end in neuronal death due to the neuroprotective action of insulin and adipocytokines. Although highly speculative, it could explain the lack of relationship between triglyceride levels and cortical thickness in the present study.

### Hyperglycemia reduces the neuroprotective action of the Ad/L ratio under IR conditions

During the progression of IR, insulin fails to suppress hepatic glucose production, but paradoxically accelerates lipid synthesis, leading to hyperglycemia and hypertriglyceridemia [55]. However, a subgroup of our participants showed elevated HOMA-IR together with normal blood glucose levels. In these individuals, insulin could inhibit hepatic gluconeogenesis by acting directly in the liver or indirectly via the pancreas, adipose tissue and/or the brain [56]. Therefore, as long as any of these insulin-signaling pathways remain unaltered, glucose levels will remain low.

Our results suggest that the balance between adiponectin and leptin may exert its neuroprotective action also under IR conditions as long as blood glucose levels remain normal. However, when glucose metabolism is disturbed, by the progression of IR among other factors, the neuroprotective effect of adipocytokines disappears, likely due to decreased glucose uptake by astrocytes, reduced energy supply to neurons, and/or increased neuroinflammation [57].

Our findings reinforce evidence linking diabetes and pre-diabetes with cognitive impairment [58–61], and go a step further by suggesting that this association may be mediated by the beneficial action of adipocytokines on the structural integrity of the prefrontal cortex, in particular the rostral anterior cingulate cortex (rACC) in the right hemisphere. This finding has important implications for cognitive ageing. Firstly, because the rACC is densely connected to other areas of the prefrontal cortex, making it suitable for carrying information across functional brain modules associated with social behavior, decision making, learning, attention and working memory [62]; and secondly, because ageing has shown to greatly affect the structural [63] and functional integrity of the rACC [64], which likely explains its central role in age-related cognitive decline [65]. On the basis of these results, we hypothesize that preserving functionality of adipose tissue could contribute to the success of cognitive ageing through the neuroprotective action of its adipocytokines on the rACC.

Modifiable lifestyle choices, such as increasing physical activity, eating healthy food, improving sleep quality, or decreasing stress appear to be the best approaches to prevent adipose tissue dysfunction [53]. However, once the adipose tissue becomes highly dysfunctional, therapeutic interventions with leptin and adiponectin may be more effective. Leptin replacement therapy has shown to have beneficial effects on the brain of patients with congenital leptin deficiency due to a mutation in the leptin gene. Its administration led to an increase in grey matter concentration in the ACC [66]. These effects were maintained for 3 years of supplementation, but reversed after a few weeks of withdrawal [67]. Leptin deficiency is also observed in anorexia nervosa and it is associated with the plethora of psychopathological, cognitive and sleep disturbances that characterize these patients. This symptomatology improved after exogenous administration of a recombinant human leptin for up to 14 days [68]. Additionally, low leptin levels could partially drive cognitive impairment in AD, not only by its action on neuronal growth and function but also by its ability to inhibit amyloidogenic pathways and reduce tau phosphorylation [57]. However, it is not yet known whether leptin therapy is able to produce a clinically relevant reduction in cognitive decline in patients diagnosed with mild cognitive impairment or AD.

The neuroprotective effect of adiponectin, on the other hand, is more likely to occur through its anti-inflammatory and insulin sensitizing roles in the brain and the periphery [69]. Recent evidence from a mouse model of amyloidosis treated with adiponectin revealed its efficacy in overcoming hippocampal synaptic disturbances and inhibiting neuronal apoptosis and inflammation [70]. Given the difficulties in producing biologically active adiponectin, lifestyle interventions and the administration of adiponectin receptor agonists and drugs capable of increasing their circulating levels are a promising alternative [71]. In this vein, leptin replacement has shown to normalize adiponectin concentrations in leptin deficient ob/ob mice, with the consequent reductions in systemic oxidative stress and inflammation [32].

### Absence of evidence for the moderating role of the remaining MtbS components on the neuroprotective action of adipocytokines

The present study does not allow us to draw strong conclusions about the impact of the remaining MtbS components on the neuroprotective action of adipocytokines because the range of Bayes factors was very close to 1, which is considered anecdotal evidence according to Lee and Wagenmakers' classification scheme [72]. In the particular case of abdominal obesity, its potential effect could be due to associated metabolic disturbances, which were included as confounding variables. A similar explanation could apply to HDL cholesterol, as triglycerides largely determine plasma levels of this lipoprotein [73]. The lack of evidence regarding the impact of hypertension is not surprising considering the inconsistencies linking hypertension to cognitive dysfunction and cognitive decline [74]. The effect of hypertension could also be masked by parallel changes affecting the Ad/L ratio, as there is evidence that both low levels of adiponectin [75] and high levels of leptin [76] may act as mediators in the development of obesity-associated hypertension.

### Limitations

Our study is subject to several limitations that warrant further consideration. Due to the cross-sectional nature of the study, it is challenging to derive causal relationships. Additionally, although all participants were cognitively normal, we cannot rule out that some of them had significant levels of AD neuropathology, especially in view of the existing associations between systemic IR and increased levels of amyloid-β42 in cerebrospinal fluid [77]. The study was further limited by the lack of a broader neuropsychological battery that included a comprehensive assessment of memory and other cognitive domains. This would not only have allowed us to assess the moderating effect of IR and other metabolic alterations on each of these cognitive domains separately, but it would also have contributed to clarify the lack of relationship between adiponectin and cognitive functioning [27]. Unfortunately, sample size and multicollinearity did not allow us to assess whether the three-way interaction was further moderated by sex and/or BMI status. Finally, we did not control for possible effects that other hormones (e.g., pancreatic, neurohypophysial and adenohypophysial among others) may have exerted on the moderating role of glycemia and triglyceridemia in the neuroprotective action of adipocytokines. All these aspects should be specifically addressed in future studies.

## CONCLUSIONS

The results derived from the present study suggest that the balance between adiponectin and leptin may be neuroprotective in ageing, although this effect is conditioned by the insulin status and other metabolic alterations. In particular, we showed that neuroprotection disappears under conditions of high insulin sensitivity when triglyceride levels are elevated. Whereas in conditions of high IR, elevated circulating glucose levels are the main limiting factor. Although the mechanisms behind these relationships need to be elucidated, results indicated that a target for the neuroprotective action of these adipocytokines, at least under conditions of IR, may be the rACC, a cortical hub linking cerebral areas governing emotion and cognitive control highly affected by ageing. Future research is required to determine whether glucose uptake as well as functional and structural connectivity patterns of this ventromedial prefrontal area are also associated with adipose tissue dysfunction, and whether interventions based either on modifiable lifestyle factors or exogenous administration of adipocytokines or drugs able to change their circulating levels improve cognition in ageing through their actions on the rACC.

## MATERIALS AND METHODS

### Participants

This cross-sectional study included 239 participants (52 to 87 years, mean±SD: 67.4±5.9, 149 females) recruited from senior citizens associations, health screening programs, and Primary Care Health Centers in Seville and Santiago de Compostela. Participants were enrolled between September 2017 and November 2019 regardless of whether or not they presented obesity, type-2 diabetes, and/or MtbS provided that they were cognitively and neurologically normal. All of them underwent neurological and neuropsychological assessments to discard objective cognitive impairment and early signs of dementia. Participants showed normal cognitive performance in the neuropsychological tests relative to appropriate reference values for age and education level, a global score of 0 in the Clinical Dementia Rating, scores ≥ 27 in the Mini Mental State Examination, and normal independent function according to the Spanish version of the Interview for Deterioration in Daily Living Activities [78]. Participants taking drugs known to affect cognition or with a history of psychiatric illnesses, addiction disorders, or severe medical conditions such as coronary heart disease, stroke, sleep apnea, infectious disease, or cancer were excluded. The presence of depression was ruled out by the shorter version of the Geriatric Depression Scale (scores ≤ 5) [79]. All participants gave informed consent to the experimental protocol approved by the Ethical Committee for Clinical Research of the Junta de Andalucía and by the Galician Clinical Research Ethics Committee (CEIC) according to the principles outlined in the Declaration of Helsinki as revised in 2008.

### Cognitive assessment

All participants were administered the shortened version of the Boston Naming Test (BNT); the semantic and letter verbal fluency tests based on the “Animal” and letter “P” naming tasks; and the two forms of the Trail Making Test (TMT-A and TMT-B). All scores were *z* transformed. In the case of TMT-A and TMT-B, we used the inverse *z*-values. Principal component analysis was applied to obtain the Spearman’s ‘*g*’ factor as an index of global cognitive function. The analysis revealed two significant factors (χ^2^ = 140.5, p < 0.001). We only retained the first one (eigenvalue 1.98), which explained 39.68% of variance in the data, due to the contribution of phonological fluency (0.79), semantic fluency (0.64), TMTA-A (0.52) and TMT-B (0.66). The second factor (eigenvalue 1.14), which explained 22.8% of variance, was not considered because it was mainly due to the contribution of BNT (0.86) and TMT-A (0.61). We used the standardized residuals to obtain the latent variable *g* (factor loading TMT-A: 0.81; TMT-B: 0.80; phonological fluency: 0.58; semantic fluency: 0.55; BNT: 0.23).

### Anthropometric, resting heart rate and blood pressure

Anthropometric examination included three body size measurements: height, weight, and waist circumference. Waist circumference was measured in standing position with non-elastic tape at the middle between the upper point of the bilateral iliac crest and the inferior margin of the rib cage in the horizontal plane at the end of expiration. The BMI was calculated by dividing weight in kilograms by the square of height in meters.

Heart rate and systolic/diastolic blood pressure measurements were obtained in sitting position, after spending 10 min in a quiet room maintained at a constant temperature of 22° C.

### Determination of cardiorespiratory fitness (CRF)

To avoid the burden of exercise testing, we used a low-risk, low-cost, non-exercise estimate of CRF initially conducted in a cohort mainly integrated by men [80] and later validated in three different cohorts aged 20 to 70 years [81] as well as in a sample of adults aged 60 to 80 years [82]. The equation-derived estimate of CRF is based on sex, age, BMI, resting heart rate, and self-reported physical activity. The self-reported physical activity was determined from a single exercise history question in which participants were asked to identify one of five physical activity categories that reflect their usual pattern of daily physical activity [81]. CRF, expressed in ml min^-1^ kg^-1^, is derived from the following equation:

CRF = Sex × 2.77 – Age × 0.1 – BMI × 0.17 – heart rate × 0.03 + self-reported physical activity + 18.07

### Biochemical determinations

Participants were instructed to fast for 12 hours prior to the blood collection appointment. One technician called them the evening before as a reminder. Upon arrival at the laboratory, they were asked about the time they had ingested their last food or drink. Fasting blood samples were taken at 9:00-10:00 AM in all participants to control for potential circadian effects. Serum levels of glucose, total cholesterol, LDL cholesterol, HDL cholesterol, and triglycerides were obtained with the automated A15 Random Access Analyzer® (Biosystems S.A., Barcelona, Spain) using Biosystems reagents. Serum insulin levels were determined with Quantikine ELISA kits (R&D Systems, Minneapolis, MN, USA) following the manufacturer’s instructions. Adiponectin and leptin levels were measured in plasma samples with Luminex human bead-based assays (Bio-Techne R&D Systems) using a Luminex platform (BioPlex 200, Bio-Rad, Hercules, California, USA), according to manufacturer's instructions. For all determinations, samples, calibrators, and controls were analyzed in duplicates, and mean values were used for statistical purposes.

HOMA-IR was calculated by dividing the product of fasting serum insulin (mU/l) and glucose (mmol/l) by 22.5. SI units for insulin were transformed to conventional units by dividing by a factor of 6.

### Definition of MtbS components

The different components of MtbS were included in the interaction term as categorical factors based on the different cut-offs established by the harmonized definition where 3 of the 5 risk factors are present [83]: abdominal obesity (waist circumference ≥94 and ≥80 cm for European men and women, respectively), hypertriglyceridemia (≥1.7 mmol/l), reduced HDL cholesterol (<1.03 mmol/l in men and <1.29 mmol/l in women), hypertension (systolic ≥130 mmHg and/or diastolic ≥85 mmHg), and hyperglycemia (≥5.6 mmol/l or previously diagnosed type-2 diabetes). Drug treatments for hypertension, type-2 diabetes, and dyslipidemia were also considered as fulfilled criteria.

### MRI acquisition and cortical thickness estimation

Structural brain images were acquired on a Philips Ingenia 3T MRI scanner equipped with a 32-channel head coil (Philips, Best, Netherlands). A whole-brain T1-weighted magnetization prepared rapid gradient echo was acquired in the sagittal plane using the following parameters: repetition time = 2600 ms, echo time = 4.7 ms, flip angle = 9°, matrix = 384 × 384, voxel resolution = 0.65 mm^3^ isotropic, and no gap between slices. Head motion was minimized by using a head restraint system and by placing foam padding around the subject's head. Participants were provided with headphones and foam earplugs to attenuate scanner noise.

Structural MRI data was processed using the pipeline of Freesurfer v6.0 (https://surfer.nmr.mgh.harvard.edu/) that involves intensity normalization, registration to Talairach, skull stripping, segmentation of cerebrospinal fluid, grey matter and white matter, tessellation of the white matter boundary, and automatic correction of topological defects. Pial surface misplacements and erroneous white matter segmentation was manually corrected on a slice-by-slice basis by one experienced technician to enhance the reliability of cortical thickness measurements, and then supervised by a senior researcher (JLC). Cortical thickness was defined as the average of the shortest distance between the pial surface and the grey matter-white matter boundary at each vertex across the cortical mantle.

### Statistical analysis

### Cognition

Before proceeding with multiple regression analyses, we applied the Yeo-Johnson transformation to the cognitive index derived from the latent variable *g* to reduce harmful effects of skewedness and heteroscedasticity on the model [84]. Secondly, the variables included in the interaction term were mean centered and transformed to *z* scores. Thirdly, for each one of the models we checked the linearity of the data, homogeneity of variance, and normality of residuals by inspecting the residuals vs. fitted plot, the spread-location plot, and the normal probability plot of residuals, respectively. We next tested multicollinearity through the variable inflation factor. We considered it acceptable if this value was below 5. And finally, we ruled out the presence of influential cases by examining the residuals vs. Leverage plot.

To evaluate whether the association between adipocytokines and cognition was moderated by the relationship between HOMA-IR and the different MetS components, we built two models, one that included the main effects and the two-way interactions between the three predictors of interest (null model) and another that further included the three-way interaction (experimental model). First, we applied the frequentist approach because it remains the dominant paradigm, and then we applied the Bayesian approach to overcome the problem of multiple comparisons and quantify the evidence for both the null and the alternative hypothesis [85]. For the former approach, we computed F-tests between the two-way and three-way interaction models; while for the latter, we compared the strength of the Bayes factor for the two-way interaction model versus the model adding the three-way interaction. We used the classification scheme proposed by Lee and Wagenmakers [72] to interpret the Bayes factor.

After inferential evidence of a main effect, we computed the standardized measure of effect size (Cohen’s f^2^), which allows determining the local effect size within the context of a multivariate regression model. As the interaction terms included at least one continuous moderator, we computed the standardized effect size (δ) by dividing the conditional effect at 1 SD below and above the mean of the moderator by the root square of the mean square residual. Values of 0.4, 1.0, and 1.6 were considered “small”, “medium”, and “large” differences, respectively [86]. Finally, to establish the precision of effect sizes, we computed 95% confidence intervals (CI_0.95_) using the normal approximated interval with bootstrapped bias and standard error through the function bootci implemented in Matlab.

### Cortical thickness

Individual cortical thickness maps were Box-Cox transformed to minimize undesirable effects such as non-additivity, non-normality and heteroscedasticity [87]. Box-Cox transformed individual cortical thickness maps were further smoothed using non-linear spherical wavelet-based de-noising schemes, which have previously shown greater specificity and sensitivity than Gaussian spatial filters for detecting local and global changes in cortical thickness [88].

The same model yielding significant results on cognition was applied to identify the influence of three-way interaction terms on cortical thickness. Results were corrected for multiple comparisons using a hierarchical statistical model that first controls the family-wise error rate at the level of clusters by applying random field theory over smoothed statistical maps (p_vertex_ < 0.001, p_cluster_ < 0.05), and next controls the false discovery rate at the level of vertex within each cluster (p < 0.05) over unsmoothed statistical maps [89].

Next, we obtained for each subject Box-Cox transformed cortical thickness values for the vertex showing the maximum T statistic within each significant cluster to estimate the standardized effect size. Results derived from the interaction model were visualized at 1 SD below and above the mean of the continuous moderator. This information was additionally used to assess the relationship between cortical thickness and cognition in isolation and in interaction with the Ad/L ratio. As the two variables of the interaction term were continuous, we computed the standardized effect size (ρ) by applying the approach suggested in [86] for moderated linear associations. Bodner and colleagues proposed that values of 0.14, 0.42, and 0.71 could be considered “small”, “medium”, and “large” differences, respectively.
